# Methylation Enables Sensitive LC-MS/MS Quantification of Ciclopirox in a Mouse Pharmacokinetics Study

**DOI:** 10.3390/molecules30173599

**Published:** 2025-09-03

**Authors:** Roshan Katekar, Zhengqiang Wang, Jiashu Xie

**Affiliations:** Center for Drug Design, College of Pharmacy, University of Minnesota, Minneapolis, MN 55455, USA; katek001@umn.edu (R.K.); wangx472@umn.edu (Z.W.)

**Keywords:** ciclopirox, LC-MS/MS, bioanalytical method validation, protein binding, pharmacokinetics

## Abstract

Ciclopirox (CPX), a topical antifungal agent of the N-hydroxypyridone class, has gained renewed interest for its potential anticancer, antiviral, antibacterial, and neuroprotective effects. However, due to lack of reliable validated bioanalytical methods, current insights into its pharmacokinetics profile beyond topical use remain limited. To support therapeutic repurposing, we developed and validated a rapid, sensitive LC-MS/MS method for systemic pharmacokinetic evaluation in mice. The method employs methyl derivatization of CPX’s N-hydroxy group, producing methylated CPX (Me-CPX) for improved chromatographic performance which was subsequently retained on the Atlantis^TM^ T3 C18 reverse phase column. Concentration of CPX is determined indirectly based on the measured response of Me-CPX. The method achieved excellent recovery, a 4-min rapid runtime, sensitivity with LLOQ of 3.906 nM (0.81 ng/mL), and a linear range up to 1000 nM (r ≥ 0.9998). All validation parameters including intra- and inter-day accuracy, precision, matrix effects, stability and dilution integrity met the criteria defined by regulatory International Council for Harmonisation (ICH) M10 bioanalytical method validation guidelines. Application of the method to in vitro plasma protein binding studies revealed high protein binding (>99%) of CPX in both human and mice plasma. Preliminary PK analysis following intravenous and oral administration in CD-1 mice demonstrated moderate systemic exposure after oral dosing, with an estimated absolute bioavailability of 52.5%. These findings establish the method’s suitability and robustness for preclinical and future clinical development of CPX as a repurposed therapeutic agent.

## 1. Introduction

**Figure 1:**
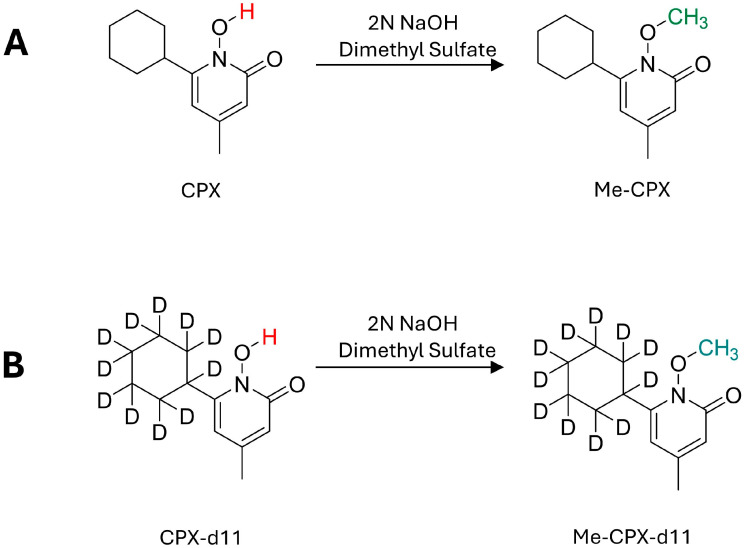
Structures and illustration for methylation reaction of (**A**) CPX; (**B**) CPX-d11 (internal standard).

Ciclopirox (CPX, Figure 1A) is a topical antifungal agent traditionally formulated as creams, gels and nail lacquers for the management of superficial mycotic infections [1,2,3,4]. It features an N-hydroxypyridone moiety known for strong metal chelation. Beyond its conventional use, CPX has garnered attention for its potential repurposing in anticancer, antiviral, antibacterial, and neuroprotective therapies. Specifically, recent studies have revealed its anticancer activity across various malignancies, including non-small cell lung cancer (NSCLC) [5,6], colorectal cancer [7], and hematological tumors [8], where CPX induces apoptosis via ER stress, impairs cellular bioenergetics, and inhibits topoisomerase IIα signaling. In NSCLC models, CPX synergizes with checkpoint kinase 1 (Chk1) inhibitors to enhance DNA damage and cell death [9]. There are studies highlighting its ability to stabilize mutant enzymes like uroporphyrinogen III synthase (UROIIIS) in congenital erythropoietic porphyria [10], and combat multidrug-resistant Gram-negative bacteria [11]. In ischemic stroke models, CPX modulates kinase pathways (AKT, GSK3β), reduces neuroinflammation, and preserves blood–brain barrier integrity [12]. Moreover, clinical investigations have begun to explore CPX’s systemic applications, including a Phase I study (NCT05647343) assessing oral CPX (ATL-001) for safety and pharmacokinetics in healthy volunteers. Recently, CPX has been shown to inhibit the replication of viruses such as SARS-CoV-2 [13], hepatitis B virus [14], and poxviruses [15]. However, despite its diverse pharmacological effects, robust data on CPX’s systemic pharmacokinetics remain limited. Comprehensive insights into its bioavailability and absorption, distribution, metabolism, and excretion (ADME) properties, particularly in murine models or humans, are currently unavailable, hindering its safe repositioning for potential systemic or non-topical applications. 

A major challenge in characterizing CPX’s pharmacokinetics is the lack of a reliable bioanalytical method. Techniques such as ultraviolet-high pressure liquid chromatography (UV-HPLC), micellar electrokinetic chromatography (MEKC), liquid chromatography-tandem mass spectrometry (LC-MS/MS), and nuclear magnetic resonance (NMR) have been utilized to quantify CPX across various matrices (see Supplementary Materials, Table S1), but often fall short in sensitivity, reproducibility, or validation. Direct chromatographic analysis of CPX is particularly challenging due to its strong interaction with silica-based stationary phases, presumably caused by the complexing properties of N-hydroxypyridone moiety, resulting in poor peak shapes or missing peaks entirely [16,17]. While ethylenediaminetetraacetic acid (EDTA) has been used as a chelating agent to mitigate these issues, reported methods exhibit limitations such as low sensitivity [18] and nonlinear calibration curve [17]. Moreover, both these methods are limited to in vitro assays and are not applied to quantitate in vivo plasma samples. Notably, our laboratory was unable to replicate the benefits of EDTA, even when using the same HPLC column cited in prior study [17]. Alternative approaches involving alkylation of the weakly acidic N-hydroxyl group have been adopted to enhance chromatographic behavior in several HPLC-based methods [16,19,20,21]. However, these strategies often lack adequate sensitivity due to UV detection limitations, demand longer run times, require higher sample volumes and frequently lack validation and application in biological matrices. Additionally, the NMR-based quantification method reported substantial error (~50%) in mouse serum without information on formal validation [22]. Other studies using mice and human plasma also failed to provide details on method development or validation, underscoring the paucity of existing bioanalytical method approaches for CPX [8,23].

Herein, we present the development and validation of a bioanalytical method incorporating a methyl derivatization step (Figure 1) to quantify CPX in mouse plasma. This validated method was subsequently applied to in vitro plasma protein binding assays and in vivo pharmacokinetic studies in mice, providing key insights into the systemic disposition of CPX and supporting its translational application in preclinical and clinical research. To our knowledge, this is the first report detailing the development of an LC-MS/MS method for CPX that has undergone comprehensive bioanalytical validation and has been applied to estimate the bioavailability of CPX specifically in mice.

## 2. Results and Discussion

### 2.1. Method Development

During method development, we initially explored the direct detection of CPX via LC-MS/MS by incorporating the metal chelator EDTA, following approaches reported in previous studies. However, these strategies, whether involving EDTA-spiked mobile phases [18] or EDTA-coated sample tubes [17], failed to improve the chromatographic performance of CPX in our lab settings, even when using the same HPLC column previously described (see Supplementary Materials, Figures S1 and S2). Therefore, we decided to adopt a reported methylation approach to mitigate the strong interaction of CPX’s N-hydroxyl group with silica-based columns [16]. The chemical conversion of CPX and its isotopically labeled internal standard (Ciclopirox-d11, CPX-d11) to their respective methylated derivatives is illustrated in Figure 1A,B. 

In this methylation process, sodium hydroxide acts as a strong inorganic base, deprotonating the N-hydroxypyridone moiety to produce a nucleophilic anion amenable to alkylation. Dimethyl sulfate serves as the methylating reagent, delivering an electrophilic methyl group that reacts with the deprotonated oxygen. Upon reaction completion, triethylamine is introduced to scavenge excess dimethyl sulfate whereby it is methylated into the quaternary methyl triethylammonium [24]. The combined use of the strongly basic sodium hydroxide and nucleophilic triethylamine ensures complete and clean conversion. The final methylated product, Me-CPX, serves as a surrogate for the parent compound and demonstrates improved chromatographic behavior on silica columns, allowing for more efficient resolution by HPLC. The internal standard CPX-d11 undergoes the same methylation reaction, resulting in Me-CPX-d11, which is quantified accordingly to ensure consistent methylation reaction and effective correction for instrument response drift over run.

#### 2.1.1. Analytical Condition Optimization and Instrumentation 

**Figure 2:**
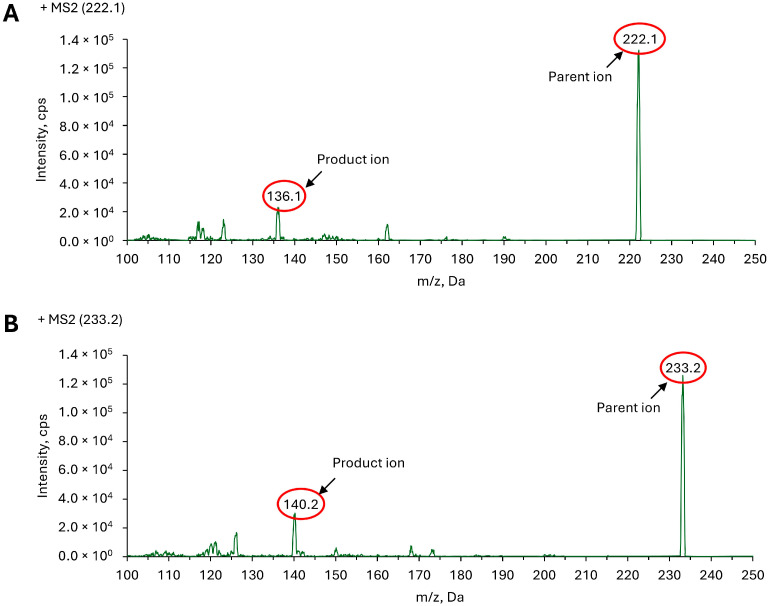
Product ion scan (MS2) for (**A**) Me-CPX; (**B**) Me-CPX-d11 showing prominent parent ion and product ion.

To optimize mass spectrometric conditions, diluted solutions of Me-CPX and Me-CPX-d11 were independently infused into the mass spectrometer using a 50:50 (*v*/*v*) mixture of water and acetonitrile containing 0.1% formic acid. Notably, methylation of CPX in acetonitrile containing 0.1% formic acid produced significantly stronger signal response compared to that in DPBS or in pure acetonitrile. Thus, the former was selected as the extraction solvent in plasma sample preparation, which is further validated by recovery experiments described below. Q1 scans were acquired in positive ionization mode to identify the predominant protonated precursor ions, which were confirmed at *m*/*z* 222.1 for Me-CPX and *m*/*z* 233.2 for Me-CPX-d11. These precursor ions were subsequently fragmented under ramped collision energy via collision-induced dissociation, yielding the product ion spectra presented in Figure 2. Optimized compound specific parameters like decluster potential, collision energy, entrance potential and collision exit potential for Me-CPX and Me-CPX-d11 were determined at 35, 35, 10, 10V and 40, 38, 10, 10V, respectively. The instrument parameters such as ion source gas 1 (GS1), ion source gas 2 (GS2), curtain gas (CUR), and collision gas (CAD) were configured at 60, 25, 40 and medium, respectively. Chromatographic conditions were also systematically optimized to achieve efficient analyte separation and reduced run times, as detailed in the Materials and Methods section.

#### 2.1.2. Procedure for Sample Preparation

Extensive optimization of the methylation reaction was performed to refine reagent amount while minimizing the use of harsh chemicals during mouse plasma sample preparation. The incubation period was systemically evaluated by sampling at defined intervals immediately following the addition of dimethyl sulfate. Linearity and reinjection reproducibility data confirmed that the optimized reagent ratios and incubation conditions (detailed below in Section 3.2.2) ensured complete CPX conversion, as no further conversion was detected even after 24 h, demonstrating excellent reaction reproducibility. Attempts to streamline the procedure by co-mixing reagents proved ineffective due to phase separation when mixing acetonitrile with sodium hydroxide or sodium hydroxide directly with dimethyl sulfate. Mixing triethylamine with dimethyl sulfate was also unsuitable, as it led to the formation of a quaternary ammonium compound that depleted the methylating agent. Notably, reactions conducted in the absence of either sodium hydroxide or triethylamine yielded markedly diminished ion signals, highlighting the necessity of both reagents in achieving efficient derivatization. Thus, the final reaction conditions described in the Material and Method section are deemed optimal for CPX conversion.

### 2.2. Bioanalytical Method Validation

#### 2.2.1. Recovery

The mean recovery values presented in Table 1 were consistent across low quality control (LQC) and high quality control (HQC) levels with precision (percent coefficient of variation, %CV) remaining within 3%, demonstrating the repeatability of the extraction process. Absolute recovery ranged from 99.77% to 101.62%, confirming that the extraction procedure was both efficient and repeatable across different levels.

#### 2.2.2. Selectivity and Sensitivity

**Figure 3:**
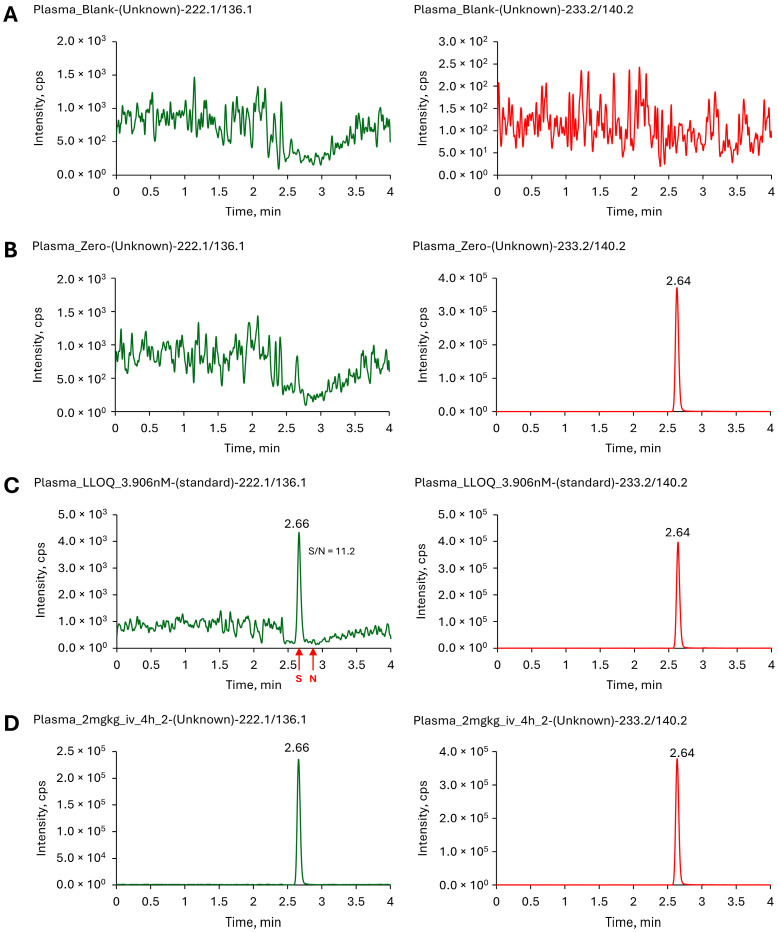
Representative multiple reaction monitoring (MRM) chromatograms for (**A**) blank mouse plasma; (**B**) blank mouse plasma sample spiked with internal standard; (**C**) mouse plasma sample at the lower limit of quantification (LLOQ); (**D**) mouse plasma sample from pharmacokinetic study. The dark green colored chromatograms on the left correspond to Me-CPX with the MRM transition *m*/*z* 222.1→136.1, while the red chromatograms on the right represent the internal standard Me-CPX-d11 with the MRM transition *m*/*z* 233.2→140.2.

The LC-MS/MS chromatograms (Figure 3) for blank, zero, lower limit of quantitation (LLOQ) samples show no interference at the retention times of the analyte and internal standard. These results demonstrated that the method is selective and free from interference, ensuring accurate quantification even at low concentration levels. The LLOQ of the method was determined to be 3.906 nM (0.81 ng/mL), where it consistently yielded a signal-to-noise (S/N) ratio greater than 10 across all replicate runs (Figure 3C) and produced a peak area at least five times higher than that observed in the blank samples. This LLOQ was further confirmed and validated through subsequent linearity and repeatability assessments (as described below), demonstrating satisfactory accuracy and precision. 

#### 2.2.3. Calibration Curve and Range

Calibration curves exhibited excellent linearity across the concentration range of 3.906–1000 nM. All non-zero calibrators (Table 2) met predefined acceptance criteria, with back-calculated concentrations exhibiting accuracies within ±15% (±20% for LLOQ) of actual concentrations and CVs below 15%. A linear regression model employing 1/x weighting provided the best fit across all runs, yielding correlation coefficients (r) consistently ≥ 0.9998 (Table 3).

#### 2.2.4. Accuracy and Precision

The method demonstrated robust accuracy and precision across all QC levels. Intra-day accuracy results (Table 4) ranged from 91.812% to 103.258%, with corresponding CVs between 0.849% and 4.667%. Inter-day accuracy (Table 5) spanned from 95.527% to 102.421%, with CVs ranging from 0.575% to 3.547%. Both intra- and inter-day performance fulfilled regulatory acceptance criteria (±15% accuracy and ≤15% precision; ±20% and ≤20% for LLOQ), confirming the method’s reliability and repeatability over time.

#### 2.2.5. Matrix Effect

Matrix effect assessments were conducted using plasma obtained from six different sources of CD-1 mice. Samples were tested at concentrations of LQC (10.417 nM) and HQC (750 nM), with three replicates per level. The observed accuracies ranged from 92.249% to 103.293%, while precision fell between 0.219% and 5.268%. All results met the established acceptance criteria, demonstrating that endogenous substances in mice plasma exert minimal influence on the quantification of CPX. A comprehensive summary of the data is available in Table 6.

#### 2.2.6. Carryover Effect

Blank samples injected immediately following the highest concentration standard displayed responses well below predefined thresholds. The analyte signal in blanks was <20% of the LLOQ signal, and the internal standard response was <5% of its signal in LLOQ samples (Table 7). These findings indicate no significant carryover, confirming the absence of cross-sample contamination. 

#### 2.2.7. Dilution Integrity

Samples with initial concentrations 200 nM and 10,000 nM were diluted 2-fold and 100-fold, respectively, to yield a final concentration 100 nM in a blank matrix. Similarly, samples with concentrations of 1500 nM and 75,000 nM underwent 2-fold and 100-fold dilutions, yielding a final concentration of 750 nM. All samples demonstrated accuracy ranging from 94.433% to 106.458%, with precision ≤4.663%. These results (Table 8) complied with the ±15% accuracy and ≤15% CV acceptance criteria, confirming the method’s robustness for handling sample dilutions from 2- to 100-fold.

#### 2.2.8. Stability Studies

As shown in Table 9, CPX demonstrated adequate stability under all tested conditions, including short-term stability (bench-top), autosampler stability, Freeze–thaw stability and long-term stability. All studies were conducted in triplicates at two levels: LQC and HQC. All stability samples met the established acceptance criteria for both accuracy (within ±15%) and precision (<15% CV).

#### 2.2.9. Reinjection Reproducibility

Reinjection of stored QC samples after 24 h in the autosampler resulted in consistent values across all QC levels (Table 10). Mean accuracy for both initial and reinjected samples was within ±15%, and precision remained ≤ 2.378%, indicating minimal degradation or analytical drift. This confirmed that the method reliably supports reanalysis of stored samples without compromising data integrity.

### 2.3. Application of Bioanalytical Method

#### 2.3.1. In Vitro Plasma Protein Binding

Plasma protein binding (PPB) of CPX was assessed in human and CD-1 mouse plasma using the rapid equilibrium dialysis (RED) system. CPX demonstrated extensive protein binding in both species, with 99.36 ± 0.02% in human plasma and 99.54 ± 0.03% in CD-1 mouse plasma. These findings indicate that the majority of CPX remains protein-bound in circulation, with minimal free drug available. The comparable binding profiles between species further supports the translational relevance of preclinical mouse data in predicting human pharmacokinetics. Additionally, the assay recovery of CPX was determined to be 102.24 ± 1.95% and 99.41 ± 0.87 in human and mouse plasma, respectively, confirming the efficiency of the dialysis system and suggesting negligible nonspecific binding to the dialysis membrane.

#### 2.3.2. In Vivo Pharmacokinetics Study

A pharmacokinetic study of CPX was performed in CD-1 mice at doses of 2 mg/kg (intravenous, IV) and 10 mg/kg (per oral, PO). Multiple vehicles were evaluated for their ability to solubilize CPX, including 1% dimethyl sulfoxide (DMSO) in DPBS and 1% DMSO in DPBS supplemented with 30% PEG300; however, these formulations failed to produce a clear solution. Solubility was successfully achieved using a 20% *w*/*v* solution of (2-Hydroxypropyl)-*β*-cyclodextrin in DPBS, which yielded a clear solution. This excipient concentration is reported as safe for both IV and PO administration in mice [25,26,27]. 

**Figure 4:**
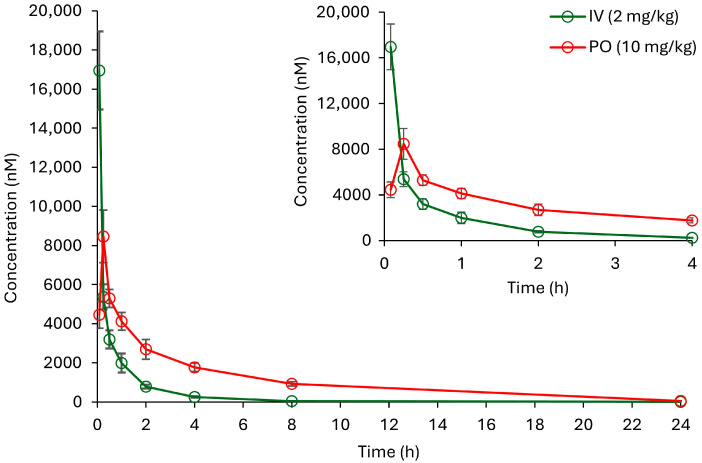
Mean plasma concentration–time profile of CPX in male CD-1 mice following a single IV and PO dose. Data represented as mean ± SD (*n* = 4).

Plasma concentrations at predetermined time points were successfully quantified using the validated LC-MS/MS method. The resulting mean plasma concentration-time curve of CPX is shown in Figure 4. Pharmacokinetic parameters were calculated using non-compartmental analysis in Phoenix WinNonlin software (version 8.5, Pharsight Corporation) and are summarized in Table 11. Following IV administration, CPX exhibited a bi-exponential decline, indicating fast distribution and elimination. Its elimination half-life (t_1/2_ = 1.5 h) was relatively short, indicating efficient systemic clearance. After oral administration, CPX reached its peak concentration (C_max_) at 0.25 h, suggesting rapid absorption from the gastrointestinal tract. In contrast to IV administration, oral dosing resulted in a notably prolonged terminal half-life (t_1/2_ = 4.1 h) and increased mean residence time (MRT_0-t_ = 4 h). The absolute oral bioavailability (%F) of CPX was estimated to be 52.522 ± 10.324%, reflecting moderate systemic exposure following oral dosing. 

## 3. Materials and Methods

### 3.1. Chemicals and Reagents

Ciclopirox (Cat no. A333509-100 mg, purity > 99%) was purchased from AmBeed, Inc., Buffalo Grove, IL, USA. Ciclopirox-d11 (Cat no. 28698) was purchased from Cayman chemicals, Ann arbor, MI, USA. Acetonitrile (Cat no. A955-4), formic acid (Cat no. A117-50) and water (Cat no. W6-4) used for chromatography were of LCMS grade and obtained from Fisher Chemical, Fair lawn, NJ, USA. Dimethyl sulfate (Cat no. D186309) and triethylamine (Cat no. 8083520100) were purchased from Sigma Aldrich, Burlington, MA, USA. Blank pooled CD-1 mouse plasma (Cat no. IGMSCD1PLAK2E50ML) and pooled human plasma (Cat no. IPLAWBK2E50ML) were purchased from Innovative research, Inc., Novi, MI, USA.

### 3.2. Method Development

#### 3.2.1. Analytical Condition Optimization and Instrumentation

Quantitative bioanalysis of CPX in murine plasma was executed utilizing hyphenated Agilent 1260 infinity chromatographic system coupled with AB Sciex QTRAP^®^ 5500 mass spectrometer (AB Sciex LLC, Toronto, ON, Canada) operated in positive electrospray ionization (ESI+) mode. Chromatographic resolution was achieved using a Waters Atlantis^TM^ T3 column (Part no. 186003734, Waters Corporation, Milford, MA, USA) with 5 cm length, 2.1 mm inner diameter and particle size of 5 µm. The mobile phase for gradient elution method consisted of aqueous 0.1% formic acid (eluent A) and acetonitrile containing 0.1% formic acid (eluent B). The gradient profile was programmed as 0.0–0.5 min (70–30% A), 0.5–1.5 min (30% A), 1.5–2.0 min (30–70% A), 2.0–4.0 min (70% A). Flow rate, column oven and autosampler temperature were maintained at 0.5 mL/min, 30 °C and 6 °C respectively. During chromatographic separation, only the eluate within the retention window of 2.4–3.0 min was selectively diverted to the mass spectrometer for detection and analysis. Multiple reaction monitoring (MRM) was used for detection and quantification of Me-CPX. The MRM analysis was conducted by monitoring the precursor ion to product ion transition of *m*/*z* 222.1/136.1 for Me-CPX and *m*/*z* 233.2/140.2 for Me-CPX-d11. System control, data acquisition, and processing were performed utilizing Analyst™ software version 1.5.2, while quantitative data analysis was conducted using MultiQuant™ software version 2.0.2.

#### 3.2.2. Procedure for Sample Preparation

The sample preparation method for CPX incorporates methylation reaction using dimethyl sulfate involves a series of steps. Calibration standards (3.906–1000 nM) and quality control samples (10.417, 83.333 and 750 nM as LQC, MQC and HQC respectively) are prepared by spiking 10 µL blank mouse plasma with 10 µL CPX stocks prepared in DMSO. The 20 µL mixture is then precipitated by 80 µL internal standard (CPX-d11, 0.1 µM) solution prepared in acetonitrile containing 0.1% formic acid. The mixture is vortexed vigorously and centrifuged at 15,000 RPM for 10 min. Supernatant (70 µL) was added with 7 µL of 2N sodium hydroxide solution followed by treatment of 7 µL of dimethyl sulfate. The reaction media was kept for incubation at 37 °C for 30 min to allow methylation of analyte and internal standard. Finally, 5 µL triethylamine was added and briefly vortexed. Samples were then transferred to vials for LC-MS/MS analysis. 

### 3.3. Bioanalytical Method Validation

The bioanalytical method validation was performed in accordance with regulatory guidelines suggested by ICH in their M10 draft which are further adopted by Food and Drug Administration (FDA) to issue guidance to industry [28]. These guidelines ensure that the validation is reliable, repeatable, and suitable for its intended purpose. The validation parameters included recovery, selectivity, calibration curve and range, accuracy and precision, matrix effect, carryover effect, dilution integrity, and stability studies. Each parameter was assessed using predefined acceptance criteria based on international guidelines.

#### 3.3.1. Recovery

Recovery was determined by comparing the peak areas of extracted QC samples with those of post-extracted blank samples spiked at equivalent concentrations. The recovery of the analyte and IS was determined in triplicates across two quality control (QC) levels such as low (10.417 nM) and high (750 nM). 

#### 3.3.2. Selectivity and Sensitivity

Selectivity was evaluated by analyzing blank biological matrix samples obtained from at least six individual sources. These samples were processed and compared with spiked samples at the lower limit of quantification (LLOQ) to confirm the absence of interfering substances at the retention times of the analyte and internal standard. The sensitivity of the method is expressed in terms of LLOQ, which was established based on a signal-to-noise ratio greater than 10 and a peak area at least five times higher than that observed in the blank sample.

#### 3.3.3. Calibration Curve and Range

Calibration curves were constructed using a series of spiked matrix samples across a predetermined concentration range of 3.906–1000 nM. A calibration curve with blank, zero and nine non-zero calibrators including LLOQ was run in three independent runs over three days. The linear regression with 1/x weighting was selected based on the best fit. Back-calculated concentrations along with mean calculated accuracy and precision are presented in results.

#### 3.3.4. Accuracy and Precision

Accuracy and precision were assessed by analyzing quality control (QC) samples at four concentration levels (LLOQ, low, medium, and high). Intra-day (within-run) and inter-day (between-run) performance was determined using replicate analyses (six replicates per level per run across three separate runs over three days). Concentrations for quality control samples were determined according to guidance (within three times of LLOQ for LQC, around geometric mean or within 30–50% of calibration curve range for MQC, and at least 75% of ULOQ for HQC).

#### 3.3.5. Matrix Effect

Matrix effects were investigated in at least six different matrix sources to avoid alteration in response due to unidentified components in different slots. The experiment was performed at two different levels, such as LQC (10.417 nM) and HQC (750 nM) in three replicates.

#### 3.3.6. Carryover Effect

Carryover was assessed by injecting blank matrix samples following the highest calibrator to avoid overestimation of analyte due to preceding samples. The response in the blank sample was required to be less than predefined acceptance criteria.

#### 3.3.7. Dilution Integrity

Dilution integrity was evaluated to ensure a reliable quantification of samples exceeding the calibration range. High analyte concentrations were diluted 2 and 100-fold with blank matrix and analyzed in five replicates at two different levels (100 nM and 750 nM). Results were corrected for dilution and assessed for accuracy and precision. Acceptable performance required values within ± 15% of expected concentrations and ≤15% variability. This confirmed the method’s suitability for quantifying diluted samples without loss of reliability.

#### 3.3.8. Stability Studies

Stability of the analyte was evaluated under various conditions including short-term (bench-top), long-term (storage), freeze–thaw, and post-preparative (autosampler) stability. All stability studies were performed at low and high concentration levels in triplicates. Bench-top stability was performed for two different time points, 2 h and 4 h. The samples were kept at ambient temperature (22 ± 2 °C) as typical experimental conditions. For freeze–thaw stability, samples were frozen at −80 ± 5 °C for 24 h and thawed to complete at least three cycles. To ensure stability of processed samples, autosampler stability was performed on stored samples for at least 24 h at 6 °C which is the set temperature for the autosampler during LC-MS/MS analysis. 

#### 3.3.9. Reinjection Reproducibility

Reinjection reliability was evaluated to confirm suitability of sample analysis after a delayed analysis or system interruption. It ensures that reanalysis due to instrument downtime or batch continuation produces valid results. Quality control (QC) samples at low, medium and high concentrations were initially injected into six replicates, then stored in the autosampler under validated conditions. After a predefined period of 24 h, the same samples were re-injected without additional processing. Consistent results between initial and reinjected data indicated the absence of degradation or system drift. Acceptance criteria required the mean accuracy of reinjected samples to be within ±15% of the nominal value. The precision, expressed as the coefficient of variation (%CV), was required to be ≤15% for each QC level.

### 3.4. Application of Bioanalytical Method

#### 3.4.1. In Vitro Plasma Protein Binding

Plasma protein binding assay was carried out in human and CD-1 mouse plasma by using rapid equilibrium dialysis device by Thermo Scientific, Durham, NC, USA (Cat no. 90006, lot no. SJ2460594). CPX at concentration of 1 µM was spiked in respective plasma (Final DMSO = 1%) and 200 µL plasma is then aliquoted into red chambers of RED plate in triplicates. 350 µL DPBS (Dulbecco phosphate-buffered saline, Gibco™, Cat no. 14190-144) was added into corresponding white chambers and allowed the drug to dialyze for 5 h at 37 °C. At the end of incubation 10 µL of post-dialysis samples (white and red chamber) were collected, matrix matched and processed as mentioned in the sample preparation method. The concentrations in 0 h sample (C_0_), post-dialysis sample in red chamber (C_p_) and post-dialysis sample in white chamber (C_b_) were determined against matrix matched calibration curve standards. The percentage of plasma protein bound (%PPB) and percentage recovery (%Recovery) were calculated using the following Equations (1) and (2) respectively:

%PPB = (C_p_ − C_b_)/C_p_ × 100 (1)

%Recovery = (C_p_ × V_p_ + C_b_ × V_b_)/(C_0_ × V_p_) × 100 (2)

where V_p_ is volume of plasma in the red chamber and V_b_ is volume of DPBS in white chamber.

#### 3.4.2. In Vivo Pharmacokinetics Study

The pharmacokinetic evaluation of CPX was conducted in male CD-1 mice (strain code: 022), sourced from Charles River Laboratories (Wilmington, MA, USA). Experimental protocol (IACUC protocol no. 2312-41621A) was approved by the Institutional Animal Care and Use Committee (IACUC) at the University of Minnesota, USA. All animal procedures adhered to IACUC policies and internal welfare guidelines, under the governance of the University of Minnesota’s Board of Regents Policy on Animal Care and Use. Animals were housed in standard conditions with controlled temperature, humidity, and a 12-h light/dark cycle, with free access to food and water. CD-1 mice were randomly divided into two groups (*n* = 4 per group) and administered CPX by intravenous (IV, 2 mg/kg) and per oral (PO, 10 mg/kg) route. The compound was dissolved in 20% (2-Hydroxypropyl)-*β*-cyclodextrin solution in DPBS (2 mg/mL for IV and 4 mg/mL for PO route respectively) to ensure appropriate solubility and dosing accuracy. Blood samples (~20 µL) were serially collected into EDTA-coated tubes from each animal at designated time points (5, 15, 30 min, and 1, 2, 4, 8, 24 h post-dose) via saphenous vein puncture. Plasma was separated by centrifugation at 3000 rpm for 15 min and stored at −80 °C until further analysis.

## 4. Conclusions

The newly developed LC-MS/MS method for CPX quantification offers notable advantages over previously reported analytical approaches. Most significantly, it delivers superior sensitivity, achieving an LLOQ of 3.906 nM (equivalent to 0.81 ng/mL), outperforming earlier methods across various matrices. The method also boasts high efficiency, with a rapid chromatographic run time of just 4 min, markedly faster than conventional HPLC methods, which can require up to 17 min per run. The method employs a linear regression model, ensuring accurate and reliable quantification across the validated concentration range, and exhibits excellent repeatability and precision, as evidenced by method validation data. In addition, it requires low plasma volume (10 µL) for sample preparation, making it particularly advantageous for studies involving limited or precious biological samples. The validated bioanalytical method has been successfully applied in both in vitro plasma protein binding assays and in vivo pharmacokinetic studies of CPX. These characteristics collectively position the validated method as a robust, sensitive, and bioanalytically sound approach for CPX determination in biological matrix.

## Figures and Tables

**Table 1:** Mean extraction recovery values for CPX and CPX-d11.

Level	CPX		CPX-d11	
	Recovery (%)	Precision (%CV)	Recovery (%)	Precision (%CV)
LQC, 10.417 nM	101.621 ± 3.006	2.958	100.742 ± 1.385	1.375
HQC, 750 nM	99.772 ± 1.278	1.281	101.378 ± 1.487	1.467

Data represented as mean ± SD (*n* = 3).

**Table 2:** Calculated concentration values, accuracy and precision data for three different calibration curve batches.

Actual Concentration (nM)	Calculated Concentration (nM)			Accuracy (%)	Precision (%CV)
	#1	#2	#3		
3.906	3.932	3.875	4.005	100.802 ± 1.668	1.655
7.813	7.247	7.747	7.610	96.438 ± 3.307	3.429
15.625	16.640	15.610	15.680	102.251 ± 3.683	3.602
31.25	31.110	31.420	31.160	99.936 ± 0.533	0.533
62.5	61.160	61.240	60.850	97.733 ± 0.330	0.337
125	128.100	127.500	128.200	102.347 ± 0.303	0.296
250	253.100	251.900	251.500	100.867 ± 0.333	0.330
500	494.700	507.600	498.000	100.020 ± 1.340	1.340
1000	1000.000	989.200	999.100	99.610 ± 0.599	0.602

#1, #2, #3 represents calculated concentration values in three different batches. Data for accuracy is represented as mean ± SD (*n* = 3).

**Table 3:** Linear regression equation parameters for three different batches.

Batch	Slope (m)	S.E. (m) ^a^	Y-intercept (b)	S.E. (b) ^b^	r
1	0.00253	1.24 × 10^−5^	0.000613	0.000774	0.99992
2	0.00253	1.27 × 10^−5^	0.000254	0.000793	0.99991
3	0.00249	8.58 × 10^−6^	0.000877	0.000536	0.99996

^a^ S.E. (m): standard error of the slope; ^b^ S.E. (b): standard error of the Y-intercept.

**Table 4:** Within-run (Intra-day) accuracy and precision values at four different QC levels.

Batch	Actual Concentration (nM)	Calculated Concentration (nM)	Accuracy (%)	Precision (%CV)
1	3.906	3.828 ± 0.146	98.007 ± 3.739	3.815
	10.417	10.710 ± 0.227	102.813 ± 2.175	2.115
	83.333	85.740 ± 0.922	102.888 ± 1.107	1.076
	750	769.417 ± 10.929	102.589 ± 1.457	1.420
2	3.906	3.586 ± 0.167	91.812 ± 4.285	4.667
	10.417	10.235 ± 0.263	98.250 ± 2.524	2.569
	83.333	85.368 ± 0.725	102.442 ± 0.870	0.849
	750	774.433 ± 7.231	103.258 ± 0.964	0.934
3	3.906	3.780 ± 0.146	96.761 ± 3.735	3.860
	10.417	10.307 ± 0.213	98.941 ± 2.045	2.067
	83.333	84.640 ± 0.815	101.568 ± 0.978	0.963
	750	760.617 ± 12.913	101.416 ± 1.722	1.698

Data represented as mean ± SD (*n* = 6).

**Table 5:** Between-run (Inter-day) accuracy and precision values at four different QC levels.

Batch	Actual Concentration (nM)	Calculated Concentration (nM)	Accuracy (%)	Precision (%CV)
1, 2, 3	3.906	3.731 ± 0.132	95.527 ± 3.389	3.547
	10.417	10.417 ± 0.125	100.001 ± 1.197	1.197
	83.333	85.249 ± 0.490	102.300 ± 0.588	0.575
	750	768.156 ± 7.356	102.421 ± 0.981	0.958

Data represented as mean ± SD (*n* = 18).

**Table 6:** Matrix effect evaluation in CD-1 mice plasma from six different sources.

Source	Actual Concentration (nM)	Calculated Concentration (nM)	Accuracy (%)	Precision (%CV)
S1	10.417	10.587 ± 0.124	101.629 ± 1.193	1.173
S2		10.377 ± 0.170	99.613 ± 1.636	1.642
S3		9.634 ± 0.508	92.487 ± 4.872	5.268
S4		9.963 ± 0.022	95.642 ± 0.209	0.219
S5		10.420 ± 0.282	100.029 ± 2.703	2.703
S6		10.760 ± 0.177	103.293 ± 1.698	1.644
S1	750	742.900 ± 12.101	99.053 ± 1.613	1.629
S2		748.367 ± 3.412	99.782 ± 0.455	0.456
S3		700.900 ± 7.749	93.453 ± 1.033	1.106
S4		691.867 ± 13.724	92.249 ± 1.830	1.984
S5		742.333 ± 4.680	98.978 ± 0.624	0.630
S6		746.033 ± 3.308	99.471 ± 0.441	0.443

Data represented as mean ± SD (*n* = 3).

**Table 7:** Determination of carryover effect in three different batches.

Batch	Me-CPX Area in LLOQ	20% of Me-CPX Area of LLOQ	Me-CPX Area in Blank	Me-CPX-d11 Area in LLOQ	5% of Me-CPX-d11 Area of LLOQ	Me-CPX-d11 Area in Blank
1	2.15 × 10^4^	4.29 × 10^3^	1.30 × 10^3^	2.03 × 10^6^	1.02 × 10^5^	6.11 × 10^2^
2	2.25 × 10^4^	4.50 × 10^3^	5.75 × 10^2^	2.24 × 10^6^	1.12 × 10^5^	3.00 × 10^2^
3	2.86 × 10^4^	5.72 × 10^3^	5.49 × 10^2^	2.64 × 10^6^	1.32 × 10^5^	4.67 × 10^2^

**Table 8:** Dilution integrity data at two levels and between 2–100 fold dilution range.

Actual Concentration (nM)	Df	Calculated Concentration (nM)	Accuracy (%)	Precision (%CV)
200	2	212.916 ± 1.805	106.458 ± 0.902	0.848
1500	2	1423.338 ± 44.739	94.889 ± 2.983	3.143
10,000	100	10,186.060 ± 95.356	101.861 ± 0.954	0.936
75,000	100	70,824.886 ± 3302.731	94.433 ± 4.404	4.663

Data represented as mean ± SD (*n* = 5).

**Table 9:** Stability study data for different experimental conditions at two different levels.

	Actual Concentration (nM)	Calculated Concentration (nM)	Accuracy (%)	Precision (%CV)
Autosampler stability (6 °C, 24 h)	10.417	10.890 ± 0.036	104.541 ± 0.346	0.331
	750	766.600 ± 6.139	102.213 ± 0.819	0.801
Bench-top stability (2 h, 22 ± 2 °C)	10.417	10.873 ± 0.240	104.381 ± 2.305	2.208
	750	756.167 ± 9.808	100.822 ± 1.308	1.297
Bench-top stability (4 h, 22 ± 2 °C)	10.417	9.735 ± 0.169	93.453 ± 1.621	1.735
	750	745.133 ± 2.702	99.351 ± 0.360	0.363
Freeze–thaw stability (−80 ± 5 °C, 3 cycles)	10.417	10.001 ± 0.365	96.010 ± 3.507	3.653
	750	746.167 ± 5.713	99.489 ± 0.762	0.766
Long-term stability (−80 ± 5 °C, 28 days)	10.417	9.656 ± 0.180	92.695 ± 1.729	1.866
	750	700.500 ± 7.134	93.400 ± 0.951	1.018

Data represented as mean ± SD (*n* = 3).

**Table 10:** Accuracy and precision values for reinjected samples after 24 h.

	Actual Concentration (nM)	Calculated Concentration (nM)	Accuracy (%)	Precision (%CV)
Injection 1	10.417	10.387 ± 0.247	99.709 ± 2.371	2.378
	83.333	83.132 ± 0.981	99.758 ± 1.177	1.180
	750	748.733 ± 6.833	99.831 ± 0.911	0.913
Injection 2, After 24 h	10.417	10.257 ± 0.182	98.466 ± 1.743	1.770
	83.333	83.013 ± 1.425	99.616 ± 1.710	1.717
	750	737.650 ± 4.504	98.353 ± 0.601	0.611

Data represented as mean ± SD (*n* = 6).

**Table 11:** Pharmacokinetic parameters of CPX in male CD-1 mice following IV and PO administration.

Pharmacokinetic Parameters	IV, 2 mg/kg	PO, 10 mg/kg
t_1/2_ (h)	1.464 ± 0.138	4.140 ± 0.287
T_max_ (h)	0.083 ± 0.000	0.250 ± 0.000
C_max_ (nM)	16,950.769 ± 1997.664	8464.616 ± 1347.941
AUC_0-t_ (h.nM)	9257.315 ± 1174.154	24,310.486 ± 4778.712
AUC_0-∞_ (h.nM)	9338.659 ± 1188.429	26,183.827 ± 2276.947
MRT_0-t_ (h)	1.024 ± 0.199	3.986 ± 0.808
V (L/kg)	2.213 ± 0.391	11.090 ± 1.489
Cl (L/h/kg)	1.046 ± 0.131	1.852 ± 0.151
Bioavailability (%F)	-	52.522 ± 10.324

Data represented as mean ± SD (*n* = 4).

## Data Availability

The raw data supporting the conclusions of this article will be made available by the authors on request.

## Supplementary Materials

The following supporting information can be downloaded at: https://www.mdpi.com/article/10.3390/molecules30173599/s1, Table S1: Summary of reported analytical and bioanalytical methods for ciclopirox; Figure S1: Representative multiple reaction monitoring (MRM) chromatograms demonstrating the lack of chromatographic enhancement for CPX using EDTA-coated sample tubes; Figure S2: Representative multiple reaction monitoring (MRM) chromatograms demonstrating the lack of chromatographic enhancement for CPX using EDTA-spiked mobile phase. References [8,16,17,18,19,20,21,22,23] are cited in the supplementary materials.

## Abbreviations

The following abbreviations are used in this manuscript:

CPX	Ciclopirox
Me-CPX	Methylated Ciclopirox
LLOQ	Lower Limit of Quantification
ICH	International Council for Harmonisation
FDA	Food and Drug Administration
PK	Pharmacokinetics
ADME	Absorption, Distribution, Metabolism, and Excretion
UV-HPLC	Ultraviolet-High Pressure Liquid Chromatography
LC-MS/MS	Liquid Chromatography-Tandem Mass Spectrometry
MEKC	Micellar Electrokinetic Chromatography
NMR	Nuclear Magnetic Resonance
EDTA	Ethylenediaminetetraacetic acid
MRM	Multiple Reaction Monitoring
QC	Quality Control
LQC	Low Quality Control
MQC	Medium Quality Control
HQC	High Quality Control
CV	Coefficient of Variation
SD	Standard Deviation
PPB	Plasma Protein Binding
IV	Intravenous
DMSO	Dimethyl Sulfoxide
IACUC	Institutional Animal Care and Use Committee
