# Supplementary material for: Methylation Enables Sensitive LC-MS/MS Quantification of Ciclopirox in a Mouse Pharmacokinetics Study
Source: Molecules. 2025 Sep 3;30(17):3599. doi: 10.3390/molecules30173599 (PMC12430005; doi:10.3390/molecules30173599)
Supplement: Supplementary file 1 [file molecules-30-03599-s001.zip › molecules-3838356-supplementary.pdf]

Supplementary materials for

Article

Methylation Enables Sensitive LC-MS/MS Quantification of Ciclopirox in a Mouse Pharmacokinetics Study

Roshan Katekar [1], Zhengqiang Wang [1] and Jiashu Xie [1,*]

[1] Center for Drug Design, College of Pharmacy, University of Minnesota, Minneapolis, MN 55455, USA;

katek001@umn.edu (R.K.); wangx472@umn.edu (Z.W.).

* Correspondence: jxie@umn.edu (J.X.); Tel.: +1 612 3016031

Table S1: Summary of reported analytical and bioanalytical methods for ciclopirox

Matrix	Instrumentation	LLOQ	Run time	Methylation treatment	Limitations in bioanalysis	Reference
In vitro nail penetration matrix	LC-MS/MS	8 ng/mL	4 min	No	Quadratic regression model applied; not reproducible in our lab.	[1]
Mouse plasma and urine	LC-MS/MS	25 ng/mL (Plasma), 250 ng/mL (Urine)	Not available	Not available	No method details or validation data reported.	[2]
WT ICR (CD-1®) outbred mice serum	NMR spectroscopy	Not available	Not available	No	Reported ciclopirox concentration shows approximately 50% error; no method validation data reported.	[3]
Human plasma	LC-MS/MS	Not available	Not available	Not available	No method details or validation data reported.	[4]
Human plasma	HPLC	20 ng/mL	12 min	Yes	Requires high sample volume (1 mL)	[5]
Absolute alcohol	Micellar electrokinetic capillary chromatography	31.3 µg/mL	6 min	No	No bioanalytical application reported.	[6]
Acetonitrile	HPLC	20 µg/mL	17 min	Yes	No method validation data and bioanalytical application reported.	[7]
Acetonitrile and water (50:50%v/v)	HPLC	0.4 µg/mL	9 min	Yes	Requires high sample volume (1 mL); no bioanalytical application reported.	[8]
5% methanol aqueous solution	HPLC	Not available	Not available	Yes	No bioanalytical application reported.	[9]

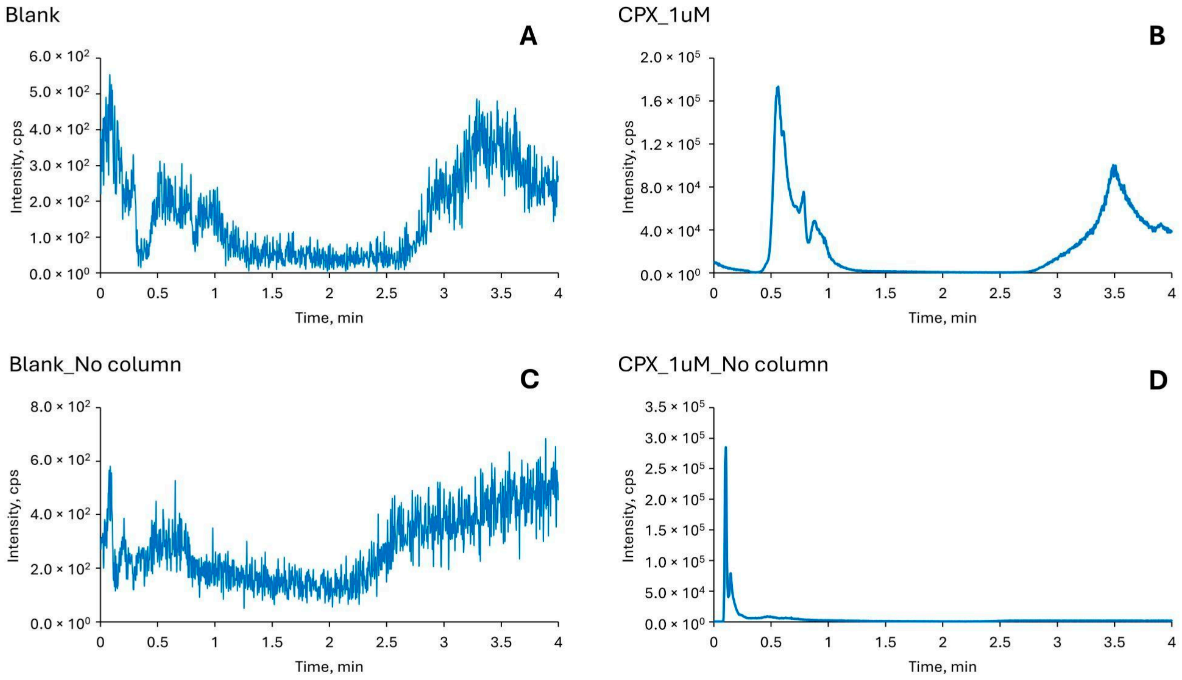

Figure S1: Representative multiple reaction monitoring (MRM) chromatograms demonstrating the lack of chromatographic enhancement for CPX using EDTA-coated sample tubes. (A) blank sample containing acetonitrile:20% ammonium hydroxide (3:1, v/v); (B) CPX at 1 μM concentration in acetonitrile:20% ammonium hydroxide (3:1, v/v); (C) blank sample containing acetonitrile:20% ammonium hydroxide (3:1, v/v) without column; (D) CPX at 1 μM concentration in acetonitrile:20% ammonium hydroxide (3:1, v/v) without column.

All samples were analyzed under the previously reported chromatographic conditions [1], employing the same Waters Atlantis® T3 column (50 mm × 2.1 mm, 5 μm) and MRM transition for CPX (m/z 208.1/136.1). Minor adjustments were made to the mass spectrometry parameters, including a declustering potential of 140 and a collision energy of 36. To demonstrate effect of EDTA spiked sample tubes, all above mentioned samples were prepared in EDTA containing tubes. For confirmation of compound presence, CPX at 1 μM concentration was injected without a column, as shown in panels (C) and (D). A prominent early peak confirmed the presence of the compound. These results confirm there is no improvement in the chromatography of CPX when using EDTA spiked sample tubes.

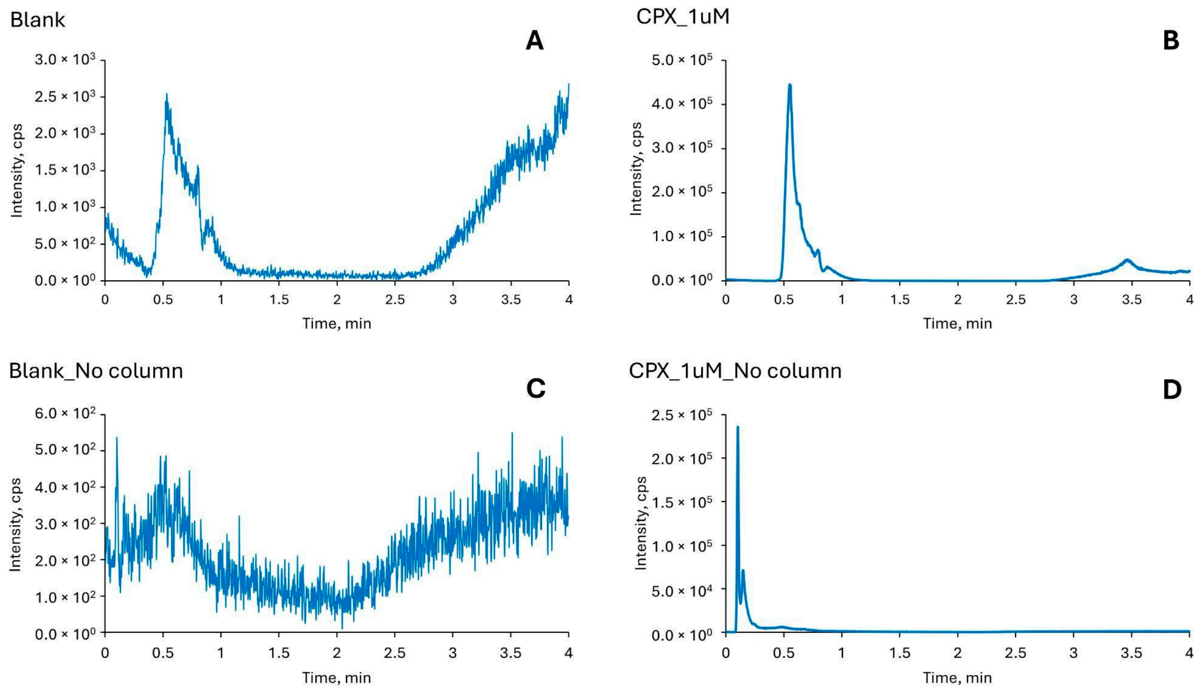

Figure S2: Representative multiple reaction monitoring (MRM) chromatograms demonstrating the lack of chromatographic enhancement for CPX using EDTA-spiked mobile phase. (A) blank sample containing acetonitrile:20% ammonium hydroxide (3:1, v/v); (B) CPX at 1 μM concentration in acetonitrile:20% ammonium hydroxide (3:1, v/v); (C) blank sample containing acetonitrile:20% ammonium hydroxide (3:1, v/v) without column; (D) CPX at 1 μM concentration in acetonitrile:20% ammonium hydroxide (3:1, v/v) without column.

Chromatographic and modified mass spectrometric parameters were applied as described in Figure S1, with minor adjustments to the mobile phase composition. To demonstrate effect of EDTA-spiked mobile phases, the mobile phase composition described in the previously reported method [1] was modified to consist of 25 μM EDTA in water (eluent A) and acetonitrile containing 0.1% formic acid (eluent B). The use of EDTA in mobile phase is suggested in another method in literature [6]. For confirmation of compound presence, CPX at 1 μM concentration was injected without a column, as shown in panels (C) and (D). A prominent early peak confirmed the presence of the compound. These results confirm there is no improvement in the chromatography of CPX when using EDTA spiked mobile phases.

References:

1. Bu, W.; Fan, X.; Sexton, H.; Heyman, I., A direct LC/MS/MS method for the determination of ciclopirox penetration across human nail plate in in vitro penetration studies. *J Pharm Biomed Anal* **2010,** 51, (1), 230-5.
2. Weir, S. J.; Dandawate, P.; Standing, D.; Bhattacharyya, S.; Ramamoorthy, P.; Rangarajan, P.; Wood, R.; Brinker, A. E.; Woolbright, B. L.; Tanol, M.; Ham, T.; McCulloch, W.; Dalton, M.; Reed, G. A.;

Baltezor, M. J.; Jensen, R. A.; Taylor, J. A., 3rd; Anant, S., Fosciclopirox suppresses growth of high-grade urothelial cancer by targeting the gamma-secretase complex. *Cell Death Dis* **2021,** 12, (6), 562.
3. Bernardo-Seisdedos, G.; Charco, J. M.; SanJuan, I.; Garcia-Martinez, S.; Urquiza, P.; Erana, H.; Castilla, J.; Millet, O., Improving the Pharmacological Properties of Ciclopirox for Its Use in Congenital Erythropoietic Porphyria. *J Pers Med* **2021,** 11, (6), 485.
4. Minden, M. D.; Hogge, D. E.; Weir, S. J.; Kasper, J.; Webster, D. A.; Patton, L.; Jitkova, Y.; Hurren, R.; Gronda, M.; Goard, C. A.; Rajewski, L. G.; Haslam, J. L.; Heppert, K. E.; Schorno, K.; Chang, H.; Brandwein, J. M.; Gupta, V.; Schuh, A. C.; Trudel, S.; Yee, K. W.; Reed, G. A.; Schimmer, A. D., Oral ciclopirox olamine displays biological activity in a phase I study in patients with advanced hematologic malignancies. *Am J Hematol* **2014,** 89, (4), 363-8.
5. Lehr, K. H.; Damm, P., Quantification of ciclopirox by high-performance liquid chromatography after pre-column derivatization. An example of efficient clean-up using silica-bonded cyano phases. *J Chromatogr* **1985,** 339, (2), 451-6.
6. Li, J.; Jiang, Y.; Sun, T.; Ren, S., Fast and simple method for assay of ciclopirox olamine by micellar electrokinetic capillary chromatography. *J Pharm Biomed Anal* **2008,** 47, (4-5), 929-33.
7. Baghel, M.; Rajput, S., Degradation and Impurity Profile Study of Ciclopirox Olamine after Pre-column Derivatization: A Risk Based Approach. *J Chromatogr Sci* **2017,** 55, (9), 899-910.
8. Escarrone, A. L. V.; Bittencourt, C. F.; Laporta, L. V.; dos Santos, M. R.; Primel, E. G.; Caldas, S. S., LC–UV method with pre-column derivatization for the determination of ciclopirox olamine in raw material and topical solution. *Chromatographia* **2008,** 67, (11), 967-971.
9. Ju-Hyun, K.; Lee, C. H.; Choi, H.-K., A method to measure the amount of drug penetrated across the nail plate. *Pharmaceutical research* **2001,** 18, (10), 1468.
